# Prediction of Tumor Cellularity in Resectable PDAC from Preoperative Computed Tomography Imaging

**DOI:** 10.3390/cancers13092069

**Published:** 2021-04-25

**Authors:** Friederike Jungmann, Georgios A. Kaissis, Sebastian Ziegelmayer, Felix Harder, Clara Schilling, Hsi-Yu Yen, Katja Steiger, Wilko Weichert, Rebekka Schirren, Ishan Ekin Demir, Helmut Friess, Markus R. Makowski, Rickmer F. Braren, Fabian K. Lohöfer

**Affiliations:** 1 Institute of Diagnostic and Interventional Radiology, School of Medicine, Technical University of Munich, 81675 Munich, Germany; friederike.jungmann@tum.de (F.J.); g.kaissis@tum.de (G.A.K.); s.ziegelmayer@tum.de (S.Z.); felix.harder@tum.de (F.H.); clara.schilling@tum.de (C.S.); marcus.makowski@tum.de (M.R.M.); 2 Department of Computing, Faculty of Engineering, Imperial College of Science, Technology and Medicine, London SW7 2AZ, UK; 3 Institute for Artificial Intelligence in Medicine and Healthcare, School of Medicine and Faculty of Informatics, Technical University of Munich, 81675 Munich, Germany; 4 Institute for Pathology, School of Medicine, Technical University of Munich, 81675 Munich, Germany; hsi-yu.yen@tum.de (H.-Y.Y.); katja.steiger@tum.de (K.S.); wilko.weichert@tum.de (W.W.); 5 Surgical Clinic and Policlinic, School of Medicine, Technical University of Munich, 81675 Munich, Germany; rebekka.schirren@tum.de (R.S.); ekin.demir@tum.de (I.E.D.); helmut.friess@tum.de (H.F.); 6 German Cancer Consortium (DKTK) Partner Site Munich, 81675 Munich, Germany

**Keywords:** pancreatic ductal adenocarcinoma, PDAC, tumor cellularity, computed tomography

## Abstract

**Simple Summary:**

Pancreatic ductal adenocarcinoma (PDAC) remains a devastating disease. However, variations in tumor biology influence individual patient outcomes greatly. We previously showed a strong association between magnetic resonance imaging-based tumor cell estimates and patient survival. In this study we aimed to transfer this finding to more broadly applied computed tomography (CT) imaging for non-invasive risk stratification. We correlated in vivo CT imaging with histopathological analyses and could show a strong association between regional Hounsfield Units (HU) and tumor cellularity. In conclusion, our study suggests CT-based tumor cell estimates as a widely applicable way of non-invasive tumor cellularity characterization in PDAC.

**Abstract:**

Background: PDAC remains a tumor entity with poor prognosis and a 5-year survival rate below 10%. Recent research has revealed invasive biomarkers, such as distinct molecular subtypes, predictive for therapy response and patient survival. Non-invasive prediction of individual patient outcome however remains an unresolved task. Methods: Discrete cellularity regions of PDAC resection specimen (*n* = 43) were analyzed by routine histopathological work up. Regional tumor cellularity and CT-derived Hounsfield Units (HU, *n* = 66) as well as iodine concentrations were regionally matched. One-way ANOVA and pairwise t-tests were performed to assess the relationship between different cellularity level in conventional, virtual monoenergetic 40 keV (monoE 40 keV) and iodine map reconstructions. Results: A statistically significant negative correlation between regional tumor cellularity in histopathology and CT-derived HU from corresponding image regions was identified. Radiological differentiation was best possible in monoE 40 keV CT images. However, HU values differed significantly in conventional reconstructions as well, indicating the possibility of a broad clinical application of this finding. Conclusion: In this study we establish a novel method for CT-based prediction of tumor cellularity for in-vivo tumor characterization in PDAC patients.

## 1. Introduction

Despite extensive therapeutic efforts and advances, pancreatic ductal adenocarcinoma (PDAC) remains a tumor entity with poor prognosis, being the fourth leading cause of cancer related death whilst accounting for only 3% of newly diagnosed cancer cases in the United States [1]. As recent developments in patient treatment have been unable to improve five-year survival above 10% [2] and incidence is increasing in developed countries, PDAC is estimated to become the second leading cause of cancer related death by 2030 [3]. This creates an urgent requirement to better understand the role of the tumor microenvironment, of possible therapeutic targets as well as of tools for patient stratification and individual outcome prediction in clinical patient care, beyond commonly applied markers of intermediate accuracy such as TNM-Stage, tumor grading or resection margin [2,4,5,6,7].

Tumor tissue composition has been a subject of intensive research due to possible implications on chemotherapy response in PDAC [8]. Previous studies identified the massive stroma formation termed desmoplasia typically observed in PDAC, mainly consisting of cancer associated fibroblasts and extracellular matrix, as promoting cancer cell proliferation and metastasis, accelerating epithelial–mesenchymal transition and hindering apoptosis in PDAC cells and have thus suggested it as a possible target for chemotherapy [8,9,10,11,12,13,14]. In other work, high tumor cellularity levels were found to be characteristics of more aggressive PDAC with poorer prognosis [15,16,17].

In recent research, pre-therapeutic image analysis has shown great success in predicting tumor characteristics such as histopathological subtypes [18], mutational status [19], specific chemotherapy response [20], and patient survival across different tumor entities [6,21,22]. This approach yields the advantages of providing no additional invasive diagnostic intervention to the patient and offering the possibility of analyzing whole-tumor characteristics. Compared to the standard approach of fine needle biopsy which captures only a small region of the tumor, image-based whole-tumor analysis may be more representative. Here we present CT-based tumor cell estimation as a non-invasive approach for in-vivo tumor characterization in PDAC patients.

## 2. Results

Overall, 139 patients diagnosed with suspected primarily resectable PDAC between September 2016 and March 2019 at our hospital were screened for eligibility. Of these patients 20 were excluded due to other pathologies (e.g., chronic pancreatitis, neuroendocrine tumors) and 79 due to unsuitable spectral CT imaging data. Thus, 43 patients were included with a mean age at diagnosis of 70.0 ± 9.8 years. Eight out of 43 patients received neoadjuvant chemotherapy, the remaining were primarily resected. The median overall survival in this cohort was 16.4 months (IQR: 5.4 months to 26.1 months). In four patients, limited metastatic disease was discovered during surgery. A summary of the clinical and histopathological characteristics of all patients included is shown in Table 1.

Patients with a PDAC containing high cellularity regions had a median overall survival of only 6.9 months (IQR: 3.6 months to 18.0 months) compared to patients exhibiting at most intermediate or low cellularity, who survived 21.8 months (IQR: 9.4 months to 26.1 months) and 22.5 months in median (IQR: 16.2 to 34.3 months), respectively. Due to the small cohort size and crossing of survival curves of the different cellularity groups no further survival analysis was performed.

To establish the relationship between histopathology-based tumor cellularity estimates and CT-derived regional HU or iodine concentration values, corresponding regions were identified in histology and image reconstructions based on surgery reports and anatomic landmarks as specified in the methods section. Table 2 shows mean HU and iodine values and 95%-confidence intervals of cellularity subgroups in conventional, monoE 40 keV and iodine map reconstructions.

**Figure 1:**
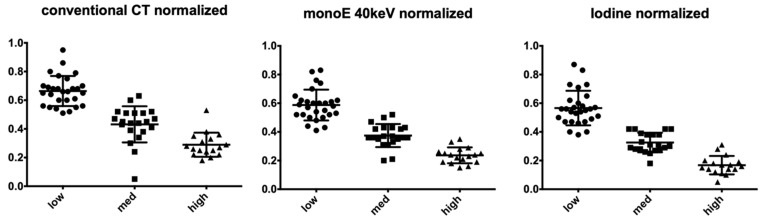
Distribution of ratio between HU values and iodine concentrations of the tumor and the aorta measured for different cellularity regions in conventional, monoE 40 keV and iodine map images.

As shown in Figure 1, normalized regional CT values of different cellularity levels showed little overlap with the least overlap observed in iodine maps. Results of the one-way ANOVA are displayed in Table 3 showing significant differences in all reconstructions and between all groups.

Pairwise *t*-tests were performed for further differentiation between the three cellularity levels in each CT reconstruction. Using the Bonferroni correction, the significance level was set to α=0.006. The results of this analysis are shown in Table 4.

**Figure 2:**
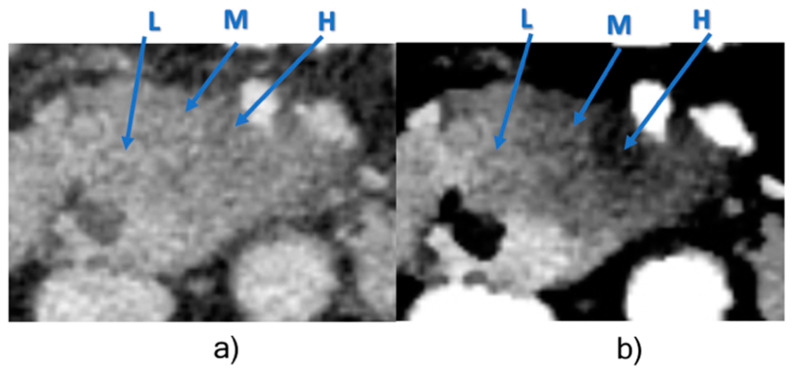
Low (L), intermediate (M) and high (H) cellularity levels within one tumor slice. (**a**) Conventional image. (**b**) monoE 40 keV image. Window settings (level/width): 50/350 for conventional CT, 250/350 for monoE 40 keV.

HU values and iodine concentrations differed between cellularity levels in all three reconstructions. However, different density regions were radiographically better delineated in monoE 40 keV images compared to conventional images due to a higher image contrast as previously shown for other tumor entities [23]. Figure 2 exemplifies this in a tumor with adjacent zones of low, intermediate and high cellularity regions in both conventional and monoE 40 keV reconstructions.

## 3. Discussion

In this study we assessed the non-invasive differentiability of PDAC tumor cellularity levels in pre-therapeutic CT images. We find a good correlation between regional CT values and tumor cellularity in conventional, virtual monoenergetic 40 keV and iodine map reconstructions, enabling excellent non-invasive discrimination between cellularity levels in all three CT reconstructions. Furthermore, our findings reinforce previous findings of high tumor cellularity as a negative predictor of patient survival.

Our results align with earlier research, finding more aggressive tumors to have a higher tumor cell proliferation rate [17,24,25] and poorer prognosis [15,17]. Other studies have suggested high stroma content and desmoplasia as the primary promoting factor of highly aggressive PDAC [8,10,14]. Targeting desmoplasia, e.g., by stroma depletion or interference with the stroma promoting Sonic Hedgehog signaling pathway, has led to prolonged survival in PDAC bearing mice [13,26]. However, PDAC in Sonic Hedgehog knockout mice show earlier metastasis and thus poorer prognosis [16,27]. Furthermore, several clinical trials targeting stroma in PDAC have shown limited efficacy [28]. These—in part contradictory—findings indicate the need for further research in this area.

To address this issue and provide new methods for patient stratification in the context of clinical research, we investigated the CT-based identification of tumor cellularity in PDAC patients. We have previously shown an inverse correlation of stroma content and percentage of tumor cells [17] and thus argue that, whereas stroma content or density may be an independent factor, tumor cellularity outweighs this effect in the context of predictive biomarkers.

Previous research has already demonstrated the possibility of non-invasive measurement of tumor cellularity in PDAC patients using ADC maps [17,29,30]. Possible advantages of MRI are the better soft tissue contrast compared to CT and thus better distinction of cystic or necrotic areas. Furthermore, additional parameters, such as intravoxel incoherent motion (IVIM) and kurtosis can be derived from diffusion weighted MRI enabling further tissue characterization [31]. However, as CT imaging is a more widely available, quantitative and standardized imaging modality for both primary diagnosis and follow up in PDAC patients, it appears more suitable for pre-treatment risk stratification and response monitoring. In our study, tumor regions with high cellularity were characterized by a lower CT attenuation in contrast-enhanced CT reconstructions. A positive correlation between the normalized contrast agent uptake and stroma component has previously been noted [17,32]. We therefore hypothesize that this finding also explains differences in HU uptake between cellularity levels that inversely correlate with stroma content.

We found tumor regions corresponding to different cellularity levels best identifiable in monoE 40 keV images due to higher contrast in pancreatic and cancer tissue as previously shown [23]. However, the HU-based identification of different cellularity level holds true for conventional images as well and 95% confidence intervals of mean HU values for each cellularity level did not overlap in conventional images neither, underlining the broad applicability of our results. For future prospective studies, possible thresholds for mean HU or iodine concentration ratio should lie outside the 95% confidence interval of the different cellularity level for each CT reconstruction. However, cut-off analyses on larger sample sizes, controlled for potential influence of different CT scanners will be required to define universally recommendable threshold values.

By exploring the possibility of non-invasive pre-treatment measurement of tumor cellularity, our work can limit undersampling effects commonly observed in biopsy-driven classification. Based on HU distribution, biopsies could be obtained in a more targeted fashion. Further prospective studies validating such an approach are required. The impact of tumor cellularity on the response to chemotherapeutic drugs is still subject to research, as promising results of pre-clinical studies investigating stroma-targeted therapy were not fully reproducible in humans so far [28]. However, in the course of development of personalized medicine techniques, non-invasive prediction of tumor cell content and thus better tumor characterization may contribute to improved individual therapy for PDAC patients.

In this study, histological analyses were performed retrospectively on H & E stained slides processed during routine histopathological work-up. Utmost care was taken to exclude any ambiguous slides resulting in the exclusion of >90% of identified slides. Nevertheless, due to the retrospective nature of the analysis and the difficulty in matching in-vivo with ex-vivo findings in soft tissue specimens mainly due to deformation, we cannot exclude the possibility of misregistration in individual cases. Furthermore, tumor cellularity was determined based on cell morphology, since no specific stainings were processed. Consequently, tumor cellularity was determined semi-quantitatively by an experienced pathologist and documented categorically rather than in absolute numbers. Herein, great care was taken to only analyze areas exhibiting a homogenous tissue composition. Despite the analysis being carried out at a high-volume center, a stringent exclusion process (i.e., scan protocol, scanner type, unequivocal correlation of in- and ex-vivo areas) led to a limited number of cases. In addition, it must be noted that we did not assess desmoplasia as an independent factor in our analysis but concentrated on tumor cellularity, as an inverse correlation of stroma and tumor cellularity has previously been shown [17,33]. Although our study has shown promising results in identifying PDAC cellularity regions in pre-therapeutic CT images as a non-invasive way of in-vivo tumor characterization, the generalizability of our findings is limited by the small cohort size and retrospective nature of our study. Due to this fact no survival analysis was performed. However, high tumor cell content has been shown to be a negative prognostic marker for overall survival in PDAC patients [15,17]. As this aspect is of high clinical interest, further investigation is required, preferably in a multi-center prospective cohort study. Furthermore, effects of individual tumor composition (for example the relative contribution of different tumor cellularity volumes) on the clinical outcome are of great interest. Such analysis was beyond the scope and possibilities within this retrospective study. Finally, the implementation of a prospective, CT-based differentiation of tumor necrosis from high cellularity areas, both presenting with low HU values, would rely on the evaluation of contrast agent up-take between the routinely acquired arterial and portal venous/parenchymal enhancement phase. Similarly, near-water attenuation and lack of contrast agent uptake would enable the exclusion of cystic lesions.

## 4. Material and Methods

The study was conducted according to Good Clinical Practice and the principles set forward in the Declaration of Helsinki. Requirements for individual written consent was waived and the study was approved by the local institutional review board of the Technical University of Munich (protocol number 180/17). Imaging and clinical data can be reviewed upon request. Third party allocation of patient data is prohibited by current regulation.

### 4.1. Patients

For this study we retrospectively analyzed imaging data, clinical data and histopathology of surgical resection specimen histopathology of 43 patients with PDAC resected between September 2016 and March 2019 in our hospital. The patient inclusion flowchart as well as the STROBE checklist are provided in the Figure S1 and Table S1. In short, 139 patients with pancreatic tumors resected between September 2016 and March 2019 were screened for eligibility. Of those, 119 patients were diagnosed with histopathologically confirmed PDAC. Twenty patients were excluded due to other pancreatic tumors. Furthermore, 79 patients were excluded due to lack of pre-treatment in-house spectral CT imaging, yielding a remaining total of 43 patients included in this study. The following clinical data were obtained for all patients using the hospital’s information system as well as the national cancer registry: Sex, age at diagnosis, tumor size (pT1/2 vs. pT3/4), lymph node status (pN0 vs. pN+), metastasis (pM0 vs. pM1), resection status (R0 vs. R+), grading (G1/2 vs. G3), type of chemotherapy received (FOLFIRINOX vs. Gemcitabine based), intention of this therapy (neoadjuvant vs. adjuvant) and overall survival time. The follow-up interval ended on 31 January 2021. 

### 4.2. Histopathological Data

Correlational analyses were retrospectively performed on archived material only. H & E-stained slides from 20 ± 5 areas of 43 specimens processed during routine histopathology work-up were collected, resulting in a total of 864 slides reviewed using a Zeiss Axioskop light microscope with a 1× objective. Of these, 62 slides were included for further analysis based on the presence of distinctive anatomical landmarks (common bile duct, main pancreatic duct, superior mesenteric artery, portal vein, splenic artery, splenic vein, duodenum, and/or spleen). Individual slide orientation was determined based on specimen ink marks applied during primary processing and the identified landmark. Corresponding areas were defined in H & E stained slides and imaging data by distance and orientation to the identified landmark in consensus reading by one experienced radiologist (FL) and one experienced pathologist (KS), resulting in 66 distinctive ROIs.

For histopathological analysis of areal tumor cellularity, one representative ROI of 1 mm^2^ was analyzed using a 40× objective. The concentration of tumor cells in this ROI was approximated without additional computer assistance by one pathologist (KS) and classified as described in previous literature [17]. In brief, tumor cellularity levels were defined as low (less than 30% tumor cells in the ROI), intermediate (30–70% malignant cells) or high (more than 70% tumor cells). For image processing, mean HU and iodine concentration of the corresponding area were defined in one ROI of 3–5 mm^2^ by one radiologist (FL). Tumor cellularity label and mean HU value were subsequently assigned for each ROI.

### 4.3. Imaging Data

**Figure 3:**
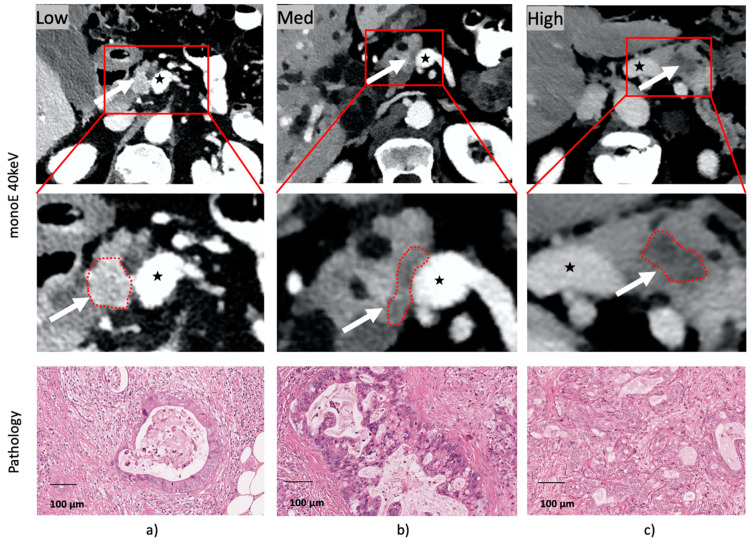
Micrographs of corresponding regions in histopathologic specimen and CT imaging for (**a**) low, (**b**) intermediate and (**c**) high cellularity level. The upper row shows the corresponding tumor region in an axial slice of the monoE 40 keV CT reconstruction. The center row depicts the zoomed in area marked in the upper row. Window settings (level/width): 50/350 for conventional CT, 250/350 for monoE 40 keV. The microscopic views of matched histopathologic cellularity regions are shown in the bottom row. Scale bar 100 µm. The tumor region in the CT images is identified by the white arrow in the upper row and by a red dotted line in the center row. The superior mesenteric vein is marked by a black star.

Pre-resection CT imaging data were retrieved from the hospital picture archiving system (PACS). All patients underwent contrast enhanced CT in the venous phase (70 s after injection of contrast agent; Ultravist^®^-370 Bayer, 70 mL, followed by a 30 mL saline chaser) using a Philips IQON Spectral CT scanner (Philips Healthcare, Best, The Netherlands). Conventional, virtual monoenergetic 40 keV images and iodine maps were reconstructed using the philips intellispace portal software (Version 11.1). Histopathological determined regions of different cellularity levels were identified in close collaboration with the responsible pathologist as described above. Corresponding HU values were measured in all images by creating a ROI with diameter approximately 5.0 mm and normalizing it to an equally sized ROI in the aorta (HU_Tumor_/HU_Aorta_). Figure 3 depicts examples of identified regions for low, intermediate and high cellularity level tumor regions in three different patients. MonoE 40 keV CT images are displayed alongside the corresponding histopathology images.

### 4.4. Statistics

All statistical analyses were performed using Python 3.8.2 and two-sided level of significance was determined to α=0.05. The D’Agostino and Pearson omnibus normality test were applied to test for normal distribution of cellularity subgroups. One-way ANOVA was used to compare the means of HU distribution of low, intermediate and high cellularity level in conventional, monoE 40 keV and iodine map images and the critical F value was set to F_2.63;0.05_ = 3.143 [34]. Paired *t*-tests were used to compare the mean HU distribution of the cellularity levels estimated from each CT reconstruction. Multiple testing correction was applied using Bonferroni correction. 

## 5. Conclusions

Here we demonstrate the feasibility of non-invasive prediction of histopathological tumor cellularity levels from pre-operative spectral CT imaging in PDAC patients. The HU-based differentiation of tumor cellularity levels was best achieved in monoE 40 keV images with high tumor cellularity regions being characterized by lower HU values. However, differentiation from conventional CT reconstructions was possible as well, suggesting wide clinical applicability of our findings, pending prospective validation in future studies.

## Figures and Tables

**Table 1:** Clinical and histopathological data of 43 patients included in the study.

Variable	Classes	N (%)
Sex	Male	21 (48.8)
	Female	22 (51.2)
Tumor size	pT1/2	21 (48.8)
	pT3/4	22 (51.2)
Lymph node status	pN0	8 (18.6)
	pN+	35 (81.4)
Metastasis	pM0	39 (90.7)
	pM1	4 (9.3)
Grading	Low grade (G1/2)	23 (53.5)
	High grade (G3)	16 (37.2)
	missing	4 (9.3)
Resection status	R0	27 (62.8)
	R+	16 (37.2)
Highest tumor cellularity level	High	17 (39.5)
	Intermediate	11 (25.6)
	Low	15 (34.9)
Chemotherapy intention	Neoadjuvant	8 (18.6)
	Adjuvant	35 (81.4)
First line chemotherapy	FOLFIRINOX	9 (20.9)
	Gemcitabine	21 (48.8)
	None or missing	13 (30.3)
Censored	Yes	26 (60.5)
	No	17 (39.5)
Overall survival	Mean (months)	18.1
	Variance (years)	13.1
Age	Mean (years)	70.0
	Variance (years)	9.8

**Table 2:** Mean HU values, iodine concentrations (normalized for aortic HU and iodine values) and 95%-CI for low, intermediate and high cellularity regions in conventional, monoE 40 keV and iodine map images.

Cellularity	Conventional CT Mean Normalized HU (95%-CI)	monoE 40keV CT Mean Normalized HU (95%-CI)	Iodine Map Mean Normalized Iodine Concentration (95%-CI)
Low cellularity	0. 66(0.62–0.70)	0.59 (0.55–0.63)	0.57 (0.52–0.61)
Intermediate cellularity	0.43 (0.37–0.49)	0.37 (0.34–0.41)	0.33 (0.29–0.36)
High cellularity	0.29 (0.24–0.33)	0.24 (0.21–0.27)	0.17 (0.13–0.20)

**Table 3:** F-statistics for normalized values of low, intermediate and high cellularity level tumor regions for conventional, monoE 40 keV and iodine map reconstructions.

Reconstruction	F-Value	*p*-Value
Conventional CT	73.01	<0.01
MonoE 40 keV CT	76.21	<0.01
Iodine maps	88.86	<0.01

**Table 4:** Pairwise *t*-test for normalized values of low, intermediate and high cellularity level tumor regions for conventional, monoE 40 keV and iodine map reconstructions, respectively.

Reconstruction	Cellularity Level	T Statistic	*p*-Value
Conventional CT	Low vs. intermediate	6.84	<0.001
	Low vs. high	13.46	<0.001
	Intermediate vs. high	4.40	<0.001
MonoE 40 keV CT	Low vs. intermediate	7.03	<0.001
	Low vs. high	11.76	<0.001
	Intermediate vs. high	5.35	<0.001
Iodine maps	Low vs. intermediate	7.66	<0.001
	Low vs. high	12.3	<0.001
	Intermediate vs. high	5.98	<0.001

## Data Availability

The data presented in this study are available on request from the corresponding author. The data are not publicly available as third party allocation of patient data is prohibited by current regulation.

## Supplementary Materials

The following are available online at https://www.mdpi.com/article/10.3390/cancers13092069/s1, Figure S1: Patient Inclusion Flowchart, Table S1: STROBE Checklist.
