# Supplementary material for: Prediction of Tumor Cellularity in Resectable PDAC from Preoperative Computed Tomography Imaging
Source: Cancers (Basel). 2021 Apr 25;13(9):2069. doi: 10.3390/cancers13092069 (PMC8123300; doi:10.3390/cancers13092069)
Supplement: Supplementary file 1 [file cancers-13-02069-s001.zip › cancers-1130535-supplementary.pdf]

cancers

MDPI

Prediction of Tumor Cellularity in Resectable PDAC from Pre-operative Computed Tomography Imaging

Friederike Jungmann, Georgios A. Kaissis, Sebastian Ziegelmayer, Felix Harder, Clara Schilling, Hsi-Yu Yen, Katja Steiger, Wilko Weichert, Rebekka Schirren, Ishan Ekin Demir, Helmut Friess, Markus R. Makowski, Rickmer F. Braren and Fabian K. Lohöfer

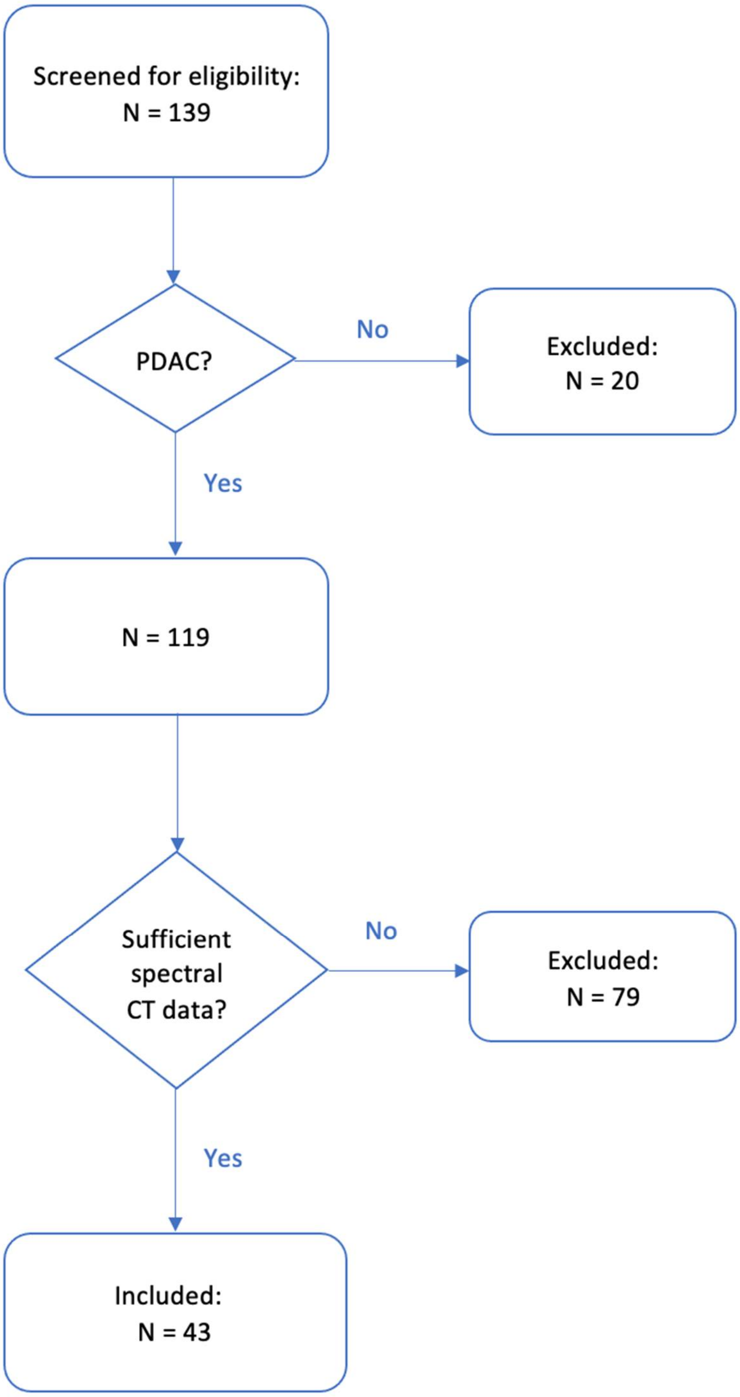

Figure S1. Patient Inclusion Flowchart.

Table S1. STROBE Checklist.

	Item No	Recommendation	Remark/ Location
**Title and abstract**	1	(*a*) Indicate the study's design with a commonly used term in the title or the abstract	Abstract
		(*b*) Provide in the abstract an informative and balanced summary of what was done and what was found	Abstract (Methods, Results)
**Introduction**			
Background/rationale	2	Explain the scientific background and rationale for the investigation being reported	Abstract, Introduction
Objectives	3	State specific objectives, including any prespecified hypotheses	Introduction, Discussion
**Methods**			
Study design	4	Present key elements of study design early in the paper	Methods
Setting	5	Describe the setting, locations, and relevant dates, including periods of recruitment, exposure, follow-up, and data collection	Methods
Participants	6	(*a*) Give the eligibility criteria, and the sources and methods of selection of participants. Describe methods of follow-up	Methods
		(*b*) For matched studies, give matching criteria and number of exposed and unexposed	Not applicable
Variables	7	Clearly define all outcomes, exposures, predictors, potential confounders, and effect modifiers. Give diagnostic criteria, if applicable	Methods, Results
Data sources/ measurement	8 *	For each variable of interest, give sources of data and details of methods of assessment (measurement). Describe comparability of assessment methods if there is more than one group	Methods
Bias	9	Describe any efforts to address potential sources of bias	Methods, Results

Study size	10	Explain how the study size was arrived at	Methods, Supplement
Quantitative variables	11	Explain how quantitative variables were handled in the analyses. If applicable, describe which groupings were chosen and why	Methods
Statistical methods	12	(*a*) Describe all statistical methods, including those used to control for confounding	Methods
		(*b*) Describe any methods used to examine subgroups and interactions	Methods
		(*c*) Explain how missing data were addressed	Not applicable
		(*d*) If applicable, explain how loss to follow-up was addressed	Not applicable
		(*e*) Describe any sensitivity analyses	Not applicable
**Results**			
Participants	13 *	(a) Report numbers of individuals at each stage of study—eg numbers potentially eligible, examined for eligibility, confirmed eligible, included in the study, completing follow-up, and analysed	Methods, Supplement
		(b) Give reasons for non-participation at each stage	Methods, Supplement
		(c) Consider use of a flow diagram	Supplement
Descriptive data	14 *	(a) Give characteristics of study participants (eg demographic, clinical, social) and information on exposures and potential confounders	Results, Table 1
		(b) Indicate number of participants with missing data for each variable of interest	Results, Table 1
		(c) Summarise follow-up time (eg, average and total amount)	Results, Table 1
Outcome data	15 *	Report numbers of outcome events or summary measures over time	Results
Main results	16	(*a*) Give unadjusted estimates and, if applicable,	Results

		confounder-adjusted estimates and their precision (eg, 95% confidence interval). Make clear which confounders were adjusted for and why they were included	
		(*b*) Report category boundaries when continuous variables were categorized	Not applicable
		(*c*) If relevant, consider translating estimates of relative risk into absolute risk for a meaningful time period	Not applicable
Other analyses	17	Report other analyses done—eg analyses of subgroups and interactions, and sensitivity analyses	Results
**Discussion**			
Key results	18	Summarise key results with reference to study objectives	Discussion
Limitations	19	Discuss limitations of the study, taking into account sources of potential bias or imprecision. Discuss both direction and magnitude of any potential bias	Discussion
Interpretation	20	Give a cautious overall interpretation of results considering objectives, limitations, multiplicity of analyses, results from similar studies, and other relevant evidence	Discussion
Generalisability	21	Discuss the generalisability (external validity) of the study results	Discussion
**Other information**			
Funding	22	Give the source of funding and the role of the funders for the present study and, if applicable, for the original study on which the present article is based	Preamble
